# Clicking in a Killer Whale Habitat: Narrow-Band, High-Frequency Biosonar Clicks of Harbour Porpoise (*Phocoena phocoena*) and Dall’s Porpoise (*Phocoenoides dalli*)

**DOI:** 10.1371/journal.pone.0063763

**Published:** 2013-05-28

**Authors:** Line A. Kyhn, Jakob Tougaard, Kristian Beedholm, Frants H. Jensen, Erin Ashe, Rob Williams, Peter T. Madsen

**Affiliations:** 1 Department of Bioscience, Aarhus University, Aarhus, Denmark; 2 Zoophysiology, Department of Bioscience, Aarhus University, Aarhus, Denmark; 3 Sea Mammal Research Unit, Scottish Oceans Institute, University of St Andrews, St Andrews, United Kingdom; CNRS, France

## Abstract

Odontocetes produce a range of different echolocation clicks but four groups in different families have converged on producing the same stereotyped narrow band high frequency (NBHF) click. In microchiropteran bats, sympatric species have evolved the use of different acoustic niches and subtly different echolocation signals to avoid competition among species. In this study, we examined whether similar adaptations are at play among sympatric porpoise species that use NBHF echolocation clicks. We used a six-element hydrophone array to record harbour and Dall’s porpoises in British Columbia (BC), Canada, and harbour porpoises in Denmark. The click source properties of all porpoise groups were remarkably similar and had an average directivity index of 25 dB. Yet there was a small, but consistent and significant 4 kHz difference in centroid frequency between sympatric Dall’s (137±3 kHz) and Canadian harbour porpoises (141±2 kHz). Danish harbour porpoise clicks (136±3 kHz) were more similar to Dall’s porpoise than to their conspecifics in Canada. We suggest that the spectral differences in echolocation clicks between the sympatric porpoises are consistent with evolution of a prezygotic isolating barrier (i.e., character displacement) to avoid hybridization of sympatric species. In practical terms, these spectral differences have immediate application to passive acoustic monitoring.

## Introduction

Toothed whales have evolved the use of a suite of different sounds for communication and echolocation. Communication signals can consist of both whistles, burst-pulsed calls (high repetition rate clicks) or patterned clicks [1]. Echolocation is conversely always based on clicks. The clicks vary in spectral properties among species and for some species even among individuals. A functional biosonar system requires clicks of high source level to detect and classify prey at ranges that allow the animal to find sufficient food. A high source level is in part obtained by high directionality. Directionality is determined by the ratio between the size of the transmitting organ and the wavelength of the projected sound [2], [3] and consequently, small echolocating animals must use higher frequencies than larger species to achieve similar directionality [4], [5]. This appears to be generally true for echolocating toothed whales. There are, however, profound differences in echolocation clicks among different families of toothed whales, and among species within some families that are not simply a result of body size, but could be related to habitat or prey specializations instead.

Similarly, a range of studies have shown that echolocation calls of Microchiropteran bats differ in signal peak frequency, bandwidth, duration and repetition rate in relation to their habitat [6]–[9]. Based on experimental testing, it has been shown that such acoustic adaptations to specific foraging niches between, for example, sympatric *Myotis* bats allow several species to have overlapping home ranges without competing for the same prey resources [10], [11]. It is expected that echolocating toothed whales may have similar adaptations with respect to acoustic niche partitioning, but very little is known about how different odontocete species may have adapted acoustically to their specific habitats.

Most delphinids (superfamily: Delphinoidae) produce short, broad-band echolocation clicks that can vary intra- and interspecifically and depending on background noise conditions [3], [12]. The exception is four groups of smaller toothed whales who share the same Narrow Band High Frequency (NBHF) click. This echolocation click is distinct from any other toothed whale click type and has evolved independently in all four groups. This raises the question of whether habitat or prey specializations exist for this signal type, as demonstrated in sympatric microchiropteran bats? The NBHF click is shared by six species of phocoenids (porpoises) [3], [13]–[16] six dolphin species in the Lissodelphininae subfamily [4], [17], [18], the pygmy sperm whale (*Kogia simus,* Owen, 1866) [19] and likely the Franciscana river dolphin (*Pontoporia blainvillei,* Gervais and d’Orbigny, 1844) [20]. The NBHF echolocation click is produced with essentially no energy below 100 kHz [21], which happens to be the upper effective hearing range of killer whales [22]. In contrast, porpoises can hear the calls and clicks of killer whales, which has been likened to an “acoustics arms race” between predator and prey [1]. This mismatch between the spectrum of NBHF clicks and killer whale hearing has led to the idea that the adaptive value of the NBHF click is to facilitate acoustic crypsis, allowing the NBHF species to echolocate and communicate without being heard by killer whales [19], [23], [24]. The NBHF species are found in almost all marine habitats; coastal, shelf and open ocean, except for densely ice filled waters, and the species overlap geographically in many places where they potentially compete for resources. One such example is the pairs of porpoise species that overlap in the Northwest Pacific, the Northeast Pacific and in South America. Based on the observed adaptations of echolocating bats to specific prey or microhabitats it may therefore be hypothesized that similar mechanisms are at play among toothed whales. More specifically, we would expect to find special acoustic adaptations among species sharing the same habitat. Such adaptations could be differences in for example source level, bandwidth or peak frequency as these parameters will determine detectability by changing the reflective properties of the prey, either by enhancing details by increasing bandwidth or by increasing the detection range in different habitats, e.g. cluttered vs. uncluttered and noisy vs. lower ambient noise by changing source level. A cluttered habitat could be a very reflective habitat such as a rocky archipelago. Slight differences in centroid frequency and bandwidth may in combination facilitate species recognition for NBHF species with very similar click properties ([4]). We therefore posed the hypotheses that 1) there would be acoustic differences between two sympatric NBHF species sharing the same habitat and 2) there would be acoustic differences between the echolocation clicks of the same NBHF species recorded in two different habitats.

To test these hypotheses we used a six-element linear hydrophone array to quantify the source parameters of NBHF clicks by recording harbour porpoises (*Phocoena phocoena*, L 1758) and Dall’s porpoises (*Phoconoides dalli,* True 1885) living sympatrically [25] in British Columbia (BC) and by recording the harbour porpoise in Denmark, where there is no overlap with other NBHF species. In BC, harbour and Dall’s porpoise’ habitat overlaps with that of fish-eating (resident) and mammal-eating (Bigg’s or transient) killer whales [26]. In this region, mammal-eating killer whales prey upon harbour porpoise, Dall’s porpoise, harbour seals, and Pacific white-sided dolphins [26], [27]. Both species have been recorded previously [15], [16], [28] in Japan, California and Denmark. However Dall’s porpoise were previously recorded with a single or two hydrophones only, by which the recorded clicks represent an unknown mixture of on- and off-axis clicks with no defined source properties, rendering comparison futile.

Secondly, we wanted to test if there are any general adaptive benefits of the NBHF click in relation to normal broad band dolphin clicks. Based on a number of assumptions, we therefore model the consequences of using the NBHF click in terms of absorption, spectral noise, bandwidth and masking noise.

We discuss the findings in light of the anti-predation theory, habitat specializations and character displacement and conclude that the two porpoise species, and possibly all NBHF species, have stereotyped signals likely to meet the dual requirements of operating an effective sonar system from a small head yielding a high directionality and at the same time minimizing the risk of detection by killer whales.

## Materials and Methods

### Recording Chain and Field Sites

Recordings were made with a linear array of six Reson TC 4034 omnidirectional hydrophones (Reson A/S, Slangerup, Denmark) with 20 m cable and a measured sensitivity of –221 dB re 1V/µPa (±2 dB) between 100 and 150 kHz. The hydrophones were calibrated in an anechoic tank both prior to and following the field recordings using a Reson 4014 hydrophone as a reference. Hydrophones were mounted horizontally in the same direction along a vertical perspex rod with 0.75 m hydrophone spacing, except between the two topmost hydrophones that were spaced 1.5 m apart. The 41 mm diameter Perspex rod was hollow and water-filled when submersed and very stiff to avoid flexing of the array during deployment.

Half-way through the field recordings in Canada, hydrophone 3 broke and provided no data for the remainder of the recordings.

In Canada, the array was suspended vertically below a buoy with the top hydrophone 2 m below the surface and the bottom hydrophone 6.5 m below the surface. In Denmark, the array was identical but with the top hydrophone 4 m below the surface. A 0.5 kg weight in the bottom kept the array vertical in the water. Signals were bandpass filtered with a 1 kHz (1 pole) high pass and a 180 kHz (4 poles) low pass filter, and amplified by 60 dB using custom made amplifiers. Signals were digitized in three National Instruments multifunction devices (USB-6251) at a sampling rate of 500 kHz per channel at 16 bits, using a common clock for triggering AD conversions in all devices. The frequency response of the recording system was flat (±2 dB) from 2 to 180 kHz.

Recordings were made in Denmark and in British Columbia, Canada. At all locations, recordings were made from a small boat with an outboard engine. Porpoises were approached at low speed and the array lowered into the water when the engine was stopped. Recordings were made over several minutes and the procedure repeated with the same or a new group. In Canada, recordings were obtained at several different sites near the Broughton Archipelago (50°36’N, 126°40’W) in July 2009. Here the hard bottom is composed of rocks covered with kelp. Harbour porpoises were primarily encountered in Beware Passage and Retreat Passage whereas Dall’s porpoises were consistently found in tidal eddies in Blackfish Sound west of Hanson Island. On all but one occasion only one species was observed at a time and no other marine mammals were observed or detected acoustically at times of recordings. On one occasion, both porpoise species were observed in the same area. Recordings from this encounter were excluded from analysis. Killer whales were observed close to the recording sites several times and on each occasion, were identified as the fish-eating ecotype, so-called ‘residents’. Recordings were made in calm weather conditions (low winds, sea state 1), but at times of Dall’s recordings there were heavy tidal currents. In Denmark, porpoises were encountered in June 2010 in the narrowest part of Little Belt (55°33’N, 9°45’E), outside Fredericia harbour and between the highway and railway bridges. The water here is deep for Danish waters, down to 80 m. The soft bottom is composed of mud and sand with no kelp. The Little Belt is heavily trafficked, which may have increased the ambient noise level. Killer whales are extremely rare in the Danish straits. There is only one documented event of a mammal-eating killer whale in Danish Waters, when a stranded killer whale in 1861 was found to contain remains of not less than 13 porpoises and 14 harbour seals in its stomach [29].

### Click Analysis

Analysis was performed only on clicks likely to have been recorded directly in front of the vocalising animal, so-called on-axis clicks. To minimize the risk of including distorted off-axis clicks in the analysis [30] we applied a set of on-axis criteria following [18] and [4]. On-axis clicks should be: i) recorded on all six (five) channels; ii) part of a scan across the array, i.e. a series of clicks closely spaced in time with rapidly varying received levels sensu [31]; iii) be the click of maximum amplitude in the scan, and iv) of maximum amplitude on one of the four (three) middle hydrophone channels; v) the direct path of the click had to be stronger than any trailing bottom or surface reflections, and vi) only one click per scan could be on-axis and included. We did not include clicks from buzzes, i.e. click trains emitted at attempts of prey capture where click repetition rate increases to some hundred clicks per second, because these clicks always have lower source levels than clicks outside the buzz [32], which would introduce unnecessary variance into the dataset. Click source properties were quantified using a series of parameters following [3] and [30] for each click accepted as being on-axis: Duration −10 dB, given by the −10 dB points down from the peak of the signal envelope (the absolute value of the analytical signal was calculated using the “hilbert” function in Matlab). Peak frequency (FPeak). Centroid frequency (FC) defined as the frequency dividing the spectrum in two halves of equal energy. −10 dB bandwidth defined as the bandwidth at −10 dB points below the spectrum peak. −3 dB bandwidth defined as the bandwidth at –3 dB points below the spectrum peak. Root-mean-square (rms) bandwidth defined as the spectral standard deviation around the centroid frequency on a linear scale. Q-rms defined as the centroid frequency divided by the rms bandwidth. Q–3 dB defined as the peak frequency divided by the −3 dB bandwidth. The power spectrum was interpolated with a factor 10.

All analyses and signal processing was performed with custom written scripts in Matlab 6.5 (*Mathworks*).

### Estimation of Source Level

The range to the vocalising animal was estimated from the time-of-arrival differences between the six hydrophones of the array, by the algorithms from [33]and [34]. Due to the over-determined design of the array, a localization error could be assessed for each localization estimate [34]. Transmission loss (TL) was estimated from the distance assuming spherical spreading loss plus frequency dependent absorption ([2]; [35]). Although assumed to be on-axis, all source levels were calculated with unknown angle from the midline of the animal, which means that source levels are likely to be underestimated to some degree and are therefore actually apparent source level, however will be referred to as source level from hereon. Apparent source level (SL) was estimated as
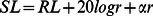
(1)where α is the absorption coefficient in dB/m and r is range in meters. The frequency-dependent sound absorption constant α was found for each species equations of [36] for the specific salinity of 33.9%o and water temperature of 9.2°C for BC and salinity of 20%o and 15°C for Denmark using the mean centroid frequency of the clicks for each species. Source levels are given as peak-peak (pp) pressure, RMS pressure and energy flux density (E) computed as follows: SL_pp_ (dB//1 µPa pp) was measured from the maximum and minimum peak pressure of the waveform. SL_RMS_ (dB//1 µPa RMS) is the rms pressure calculated over the duration_-10dB_ of the signal. SL_EFD_ (dB//1 µPa^2^s) is the signal energy integrated over the duration_-10dB_ ([30]).

The accuracy of the array localization has previously been evaluated ([4]) and based on this only clicks from animals localized within 65 m of the array where the rms-error on the transmission loss is <3 dB were used.

### Estimation of Beam Pattern

When a click from an animal at a known distance from the array is recorded simultaneously by all six hydrophones and with one hydrophone deemed to be on-axis (as by the criteria above), the angle from the midline of the animal to the line to each of the five other hydrophones can be calculated. From these angles and the received levels at the off-axis hydrophones a vertical beam pattern may be estimated (see [4]). Beam patterns were calculated for on-axis clicks recorded from animals within 20 m of the array.

### Species Discrimination Based on Echolocation Click Parameters

We used a canonical discriminant analysis in Systat 10 (SPSS Inc.) to examine the differences in source parameters among the three porpoise groups. We used the spectral properties centroid frequency and rms-bandwidth along with duration as variables. To assess whether differences between the two species could be perceptible to porpoises from British Columbia, we created a new dataset for each of the two species. All on-axis clicks and the clicks recorded simultaneously on the five other hydrophones where filtered using a filter that emulated the audiogram of a harbour porpoise [37], [38] fig. 6, in order to make the clicks resemble what the porpoises are likely to hear. The filter was made by convolving each click with an inverse-transformed interpolated linear version of the audiogram. We thus assumed that Dall’s porpoise would have the same audiogram as a harbour porpoise. After the filtering we calculated all click source parameters again for both on- and off-axis clicks and we then performed a Monte Carlo simulation where we randomly selected 100 sets of click pairs of 1, 2, 4, 8, 16 or 32 clicks for each species from the new datasets containing one on-axis click for every five off-axis clicks. The separation criterion was based on mean centroid frequencies using a ROC curve and found to 139 kHz. Click sets with mean centroid frequency below 139 kHz classified as Dall’s porpoise, above 139 kHz as harbour porpoise) the total proportion of correct classifications were calculated. The procedure was repeated ten times, allowing for calculation of standard deviations of the performance.

### Model of Target Detection Range for Signals of Different Centroid Frequency

NBHF clicks are different from typical dolphin clicks by the narrow bandwidth and by the high frequency of 130 kHz. Dolphin clicks are typically about three times wider and thus with energy spread over a much larger frequency range. These different physical properties are constraints affecting detectability and detection range, and looking in detail at these differences may shed light on the evolution of the NBHF click.

To compare the physical properties of NBHF clicks versus broadband dolphin clicks we therefore made a model to explore the consequences of the trade-off between the relative effects of 1) the possible low-noise window at 100–150 kHz in the sea [2], 2) the lower detection threshold effects of a narrow bandwidth, longer duration signal and 3) the negative effects of increased absorption with increasing frequency.

First we assumed a single signal of fixed bandwidth (15 kHz) resembling a NBHF click and compared the effects of varying the centroid frequency on absorption for the same source energy flux density. Since the ear operates as an energy detector [3], [39] different click durations with the same peak pressure will yield different detection thresholds because the energy content of a click increases with click duration. To simplify the model we therefore expressed source level as the energy flux density (dB re 1 µPas^2^). Thus a NBHF click with a duration of 100 µs and a source sound pressure level of 200 dB re 1 uPa (pp) will have the same source energy flux density as a dolphin style click with a duration of 25 µsec and a source sound pressure level of 206 dB re 1 uPa (pp) due to the longer duration. Secondly, we included a click with a three times wider bandwidth (45 kHz) resembling a dolphin style click [3] and again we varied the centroid frequency to test the effects of the increased bandwidth of a dolphin style click compared to a NBHF for each centroid frequency and for the same source energy flux density.

The model is based on the following assumptions:

1. Masking noise (NL) was estimated as *NL* = *No(F_c_)*+10log (*BW_RMS_)* [31], where *N_o_* is the background spectral noise level at the centroid frequency, *F_c_*. *N_o_* was extracted from figure 7.5 in [2] for deep water. BW_RMS_ is the rms-bandwidth of the emitted click/returning echo and was kept constant at 15 kHz to represent a NBHF click or at 45 kHz to resemble a dolphin style click.
2. Source energy flux density level (SL), receiving directivity index (DI) and target strength (TS) were assumed constant.
3. Detection threshold (DT) for the animal was assumed to be at the same echo-to-noise ratio (ENR) level above the masking noise for all centroid frequencies.
4. That the spectral noise is lowest in the environment around 130 kHz.

During transmission back and forth to the target, the energy flux density of an echolocation click is reduced as a result of absorption and geometric spreading. This reduced fraction is termed transmission loss (TL). Absorption increases with frequency and the absorption coefficient, α, can be estimated from [36] for a given frequency, water temperature and salinity. The centroid frequency of a signal thus affects the received level of the returning echo, everything else being equal, and the centroid frequency and bandwidth will influence the masking noise level, which will affect the detection range of a given target keeping all other things equal. This is formalized in the active sonar equation:
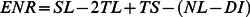
(2)where transmission loss is *TL = *20log(*r*)+α(r), r is range to target in meters and α is the absorption coefficient in dB/m at the centroid frequency of the signal. NL is noise level, TS is target strength, SL is source level and DI is directivity index. For detection of a signal of a given frequency the echo level has to exceed the noise level with some factor (ENR) by which the detection threshold (DT) can be defined as a certain dB level of ENR above the masking noise level at the centroid frequency of the echo. Thus, the echo-to noise ratio goes up when source level, target strength and directivity index go up or when transmission loss or bandwidth goes down. For the model it is assumed that ENR for detection on a statistical basis is the same for all centroid frequencies. In the model, source level receiving directivity index and target strength were assumed to be constant (assumption 2), while transmission loss and noise level were varied (as a function of varying bandwidth) and thus changing ENR. To see these changes most easily the resulting changes in ENR were normalized relative to the ENR of a NBHF signal at detection threshold.

## Results

### Canadian Porpoises

**Figure 1:**
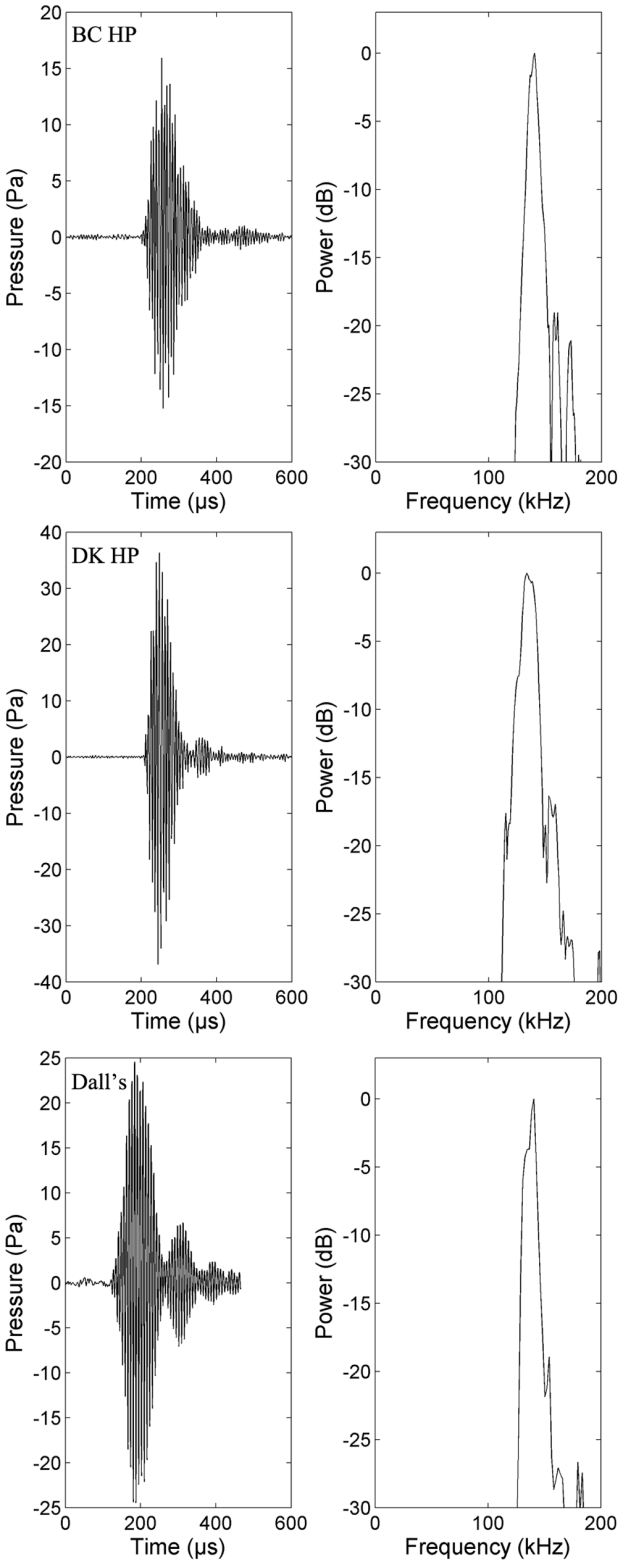
Representative clicks from a) Canadian harbour porpoise, b) Danish harbour porpoise and c) Dall’s porpoise. (Fast Fourier transform size of 512, spectrum interpolated with a factor 10, sampling rate 500 kHz, rectangular window). Note that the scale of the Y-axis in the first panel varies due to differences in received level.

Porpoises were encountered in small groups of 3–8 animals and a total of 4.7 hours and 4.5 hours of recordings were obtained from several groups of Dall’s and harbour porpoises, respectively, over several days. Of the thousands of clicks recorded, 98 clicks from Dall’s porpoise were accepted as on-axis according to the five criteria and 78 of the BC harbour porpoise clicks were classified as on-axis. The source parameters of Dall’s and harbour porpoises are summarised in table 1. The mean source level of Dall’s porpoises was 187±7 dB re 1 µPa (peak-peak). The mean centroid frequency was 137±3 kHz and the mean rms-bandwidth 8±2 kHz yielding a mean rms Q value of 17±5. For Canadian harbour porpoises the mean source level was 178±4 dB re 1 µPa (peak-peak). The mean centroid frequency was slightly higher of 141±2 kHz while the mean rms-bandwidth was similar of 8±2 kHz yielding a mean rms-Q value of 18±4. Representative clicks of the two species are shown in figure 1.

**Table 1:** Echolocation click source parameters of on-axis clicks from Danish and Canadian harbour porpoises (Phocoena phocoena) and Canadian Dall’s porpoise (Phocoenoides dalli) recorded with a six element hydrophone array.

	BC Dall’s porpoise		BC Harbour porpoise		Danish harbour porpoise	
	Phocoenoides dalli		Phocoena phocoena		Phocoena phocoena	
Parameters	Mean values ± St.d.	Range	Mean values ± St.d.	Range	Mean values ± St.d.	Range
**10dB duration, us**	104±37	53–251	88±29	48–189	54±8	35–98
**Source level, dB re 1 uPa (p.-p.)**	183±7	153–203	178±4	170–189	189±5	169–199
**Source level_-10db_, dB re 1 uPa (rms)**	172±7	141–192	166±4	158–178	178±5	158–188
**Energy flux density_-10db_ dB re 1 uPa^2^s**	132±7	104–150	125±4	116–137	135±5	114–144
**Peak frequency, kHz**	137±4	119–143	140±1	137–143	137±6	112–145
**Centroid frequency, kHz**	137±3	121–147	141±2	138–148	136±3	126–144
**3dB bandwidth, kHz**	11±5	3–23	8±3	3–19	17±5	5–36
**RMS bandwidth, kHz**	8±2	5–14	8±2	5–14	10±2	6–17
**Q_-3dB_**	15±8	6–45	20±7	7–42	9±3	3–30
**Q_RMS_**	17±4	10–29	18±4	9–28	14±3	8–25
**Directivity index, dB** *	25		24		25.6	
**Equivalent aperture, diameter, cm.** *	10		12		10	
**n**	98		77		246	

* All clicks used for vertical beam pattern estimations were recorded within 20 m from the array resulting in 5 Canadian and 19 Danish harbour porpoise and 15 Dall’s porpoise clicks.

st.d. denotes standard deviation. All other abbreviations appear in the text.

### Danish Harbour Porpoises

Harbour porpoises were recorded in Little Belt, Denmark, over three days. Animals were found in groups of sometimes more than 10 animals, actively foraging and observed together with gulls diving vigorously where the porpoises were surfacing. In total, 4.1 hours of recordings were obtained and 247 clicks fulfilled the on-axis criteria. Source parameters are summarised in table 1. Danish harbour porpoises had a mean source level of 189±5 dB re 1 µPa (peak-peak). The mean centroid frequency was 136±3 kHz and the mean rms-bandwidth was 10±2 kHz yielding a mean rms-Q value of 14±3. A representative click is shown in figure 1.

Centroid frequency, rms-bandwidth and source level was compared among the three data sets with Kruskall-Wallis One-Way Analysis of Variance on ranks. The source level of Danish harbour porpoises were significantly higher than source levels of Dall’s and BC harbour porpoises (*p*<0.001, n = 421) and BC harbour porpoises produced clicks of the lowest source levels (*p*<0.001, n = 421) among the porpoises. Clicks of BC harbour porpoises had the highest centroid frequency (*p*<0.001, *n* = 421) among the porpoises and Dall’s porpoise had the narrowest bandwidth (*p*<0.001, *n* = 421). Mann-Whitney Rank Sum Test showed that BC harbour porpoises produced clicks with energy concentrated in a narrower and higher placed frequency band than Danish harbour porpoises: Centroid frequency was significantly higher (*p*<0.001) and bandwidth significantly narrower (*p*<0.001) than for Danish harbour porpoises.

### Species Separation Based on Click Parameters

**Figure 2:**
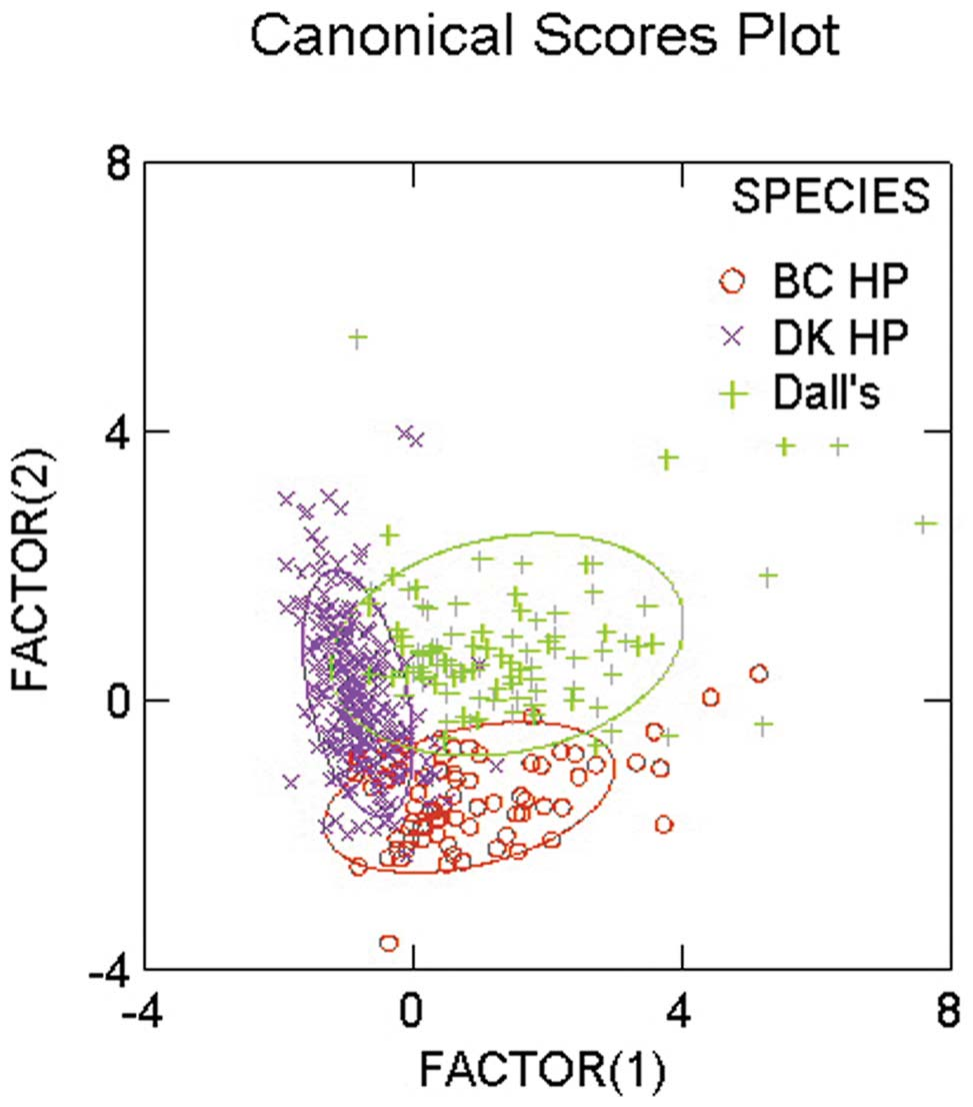
Discriminant analysis. Centroid frequency, bandwidth (rms) and duration were used to separate BC harbour porpoises, Danish harbour porpoises and Dall’s porpoises. All parameters were significantly different across populations. The three species could be separated 84% correctly.

**Figure 3:**
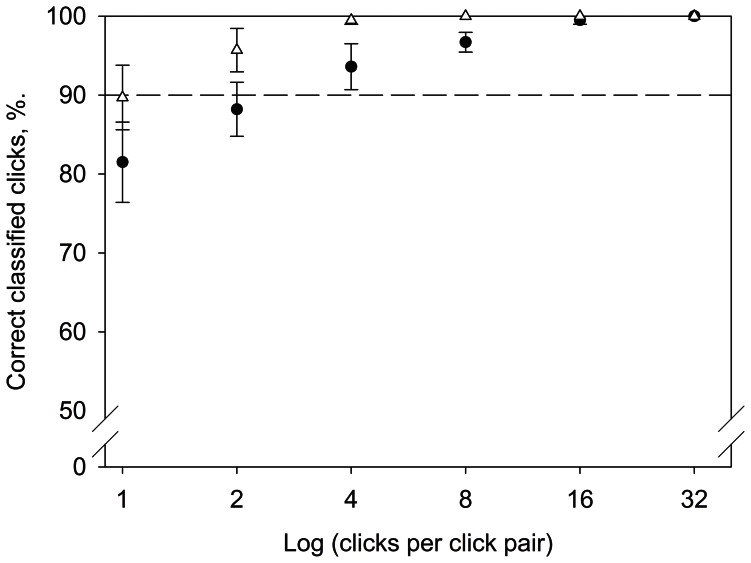
Acoustic species discrimination. Dall’s (circles) and BC-harbour porpoises (triangles) can be separated by means of differences in centroid frequency using a criterion of 139 kHz in a Monte Carlo simulation. The clicks were first filtered with the harbour porpoise’ audiogram (see text) to simulate porpoise reception. The dashed line indicates 90% correctly classified clicks. Such differences may also be useful in passive acoustic monitoring, provided there is fine-scale frequency resolution in the PAM dataloggers. The percentage correct (y-axis) for each click pair is the mean of ten rounds of randomly drawing 100 click pairs consisting of N clicks per pair (x-axis), and the values are shown with the standard error of the mean. The clicks included are one on-axis click for each five off-axis clicks.

A canonical discriminant analysis showed that the on-axis clicks from the three porpoise populations could be separated with overall 84% correct classifications based on source parameters (Table 2) for single clicks. Dall’s porpoise were equally likely to be misclassified as either BC harbour porpoise or Danish harbour porpoise. BC harbour porpoises were more often misclassified as Danish harbour porpoises than as Dall’s porpoise, while Danish harbour porpoises were more often misclassified as Dall’s porpoises (Table 2, Figure 2). Because the discriminant analysis showed good character agreement within each species, we used a Monte Carlo simulation to test whether the differences in source parameters could be used for acoustic species identification. The Monte Carlo simulation showed that Dall’s and BC harbour porpoises could be separated based on mean centroid frequency of eight randomly drawn clicks from a mixture of on-and off-axis origin with more than 90% correctly classified click pairs. When using clicks filtered with the harbour porpoise audiogram the percentage rose to more than 96% correctly classified click pairs (Figure 3).

**Table 2:** Classification matrix (cases in rows, categories classified into columns) of the canonical discriminant analysis for the three porpoise groups.

	BC HP	DK HP	Dall's	% correct
**BC harbour porpoise**	65	8	4	84
**DK harbour porpoise**	28	215	3	87
**Dall's harbour porpoise**	5	20	73	74
**Total**	98	243	80	84

Included variables are Duration_-3dB_, Centroid frequency and rms-bandwidth.

### Beam Patterns

**Figure 4:**
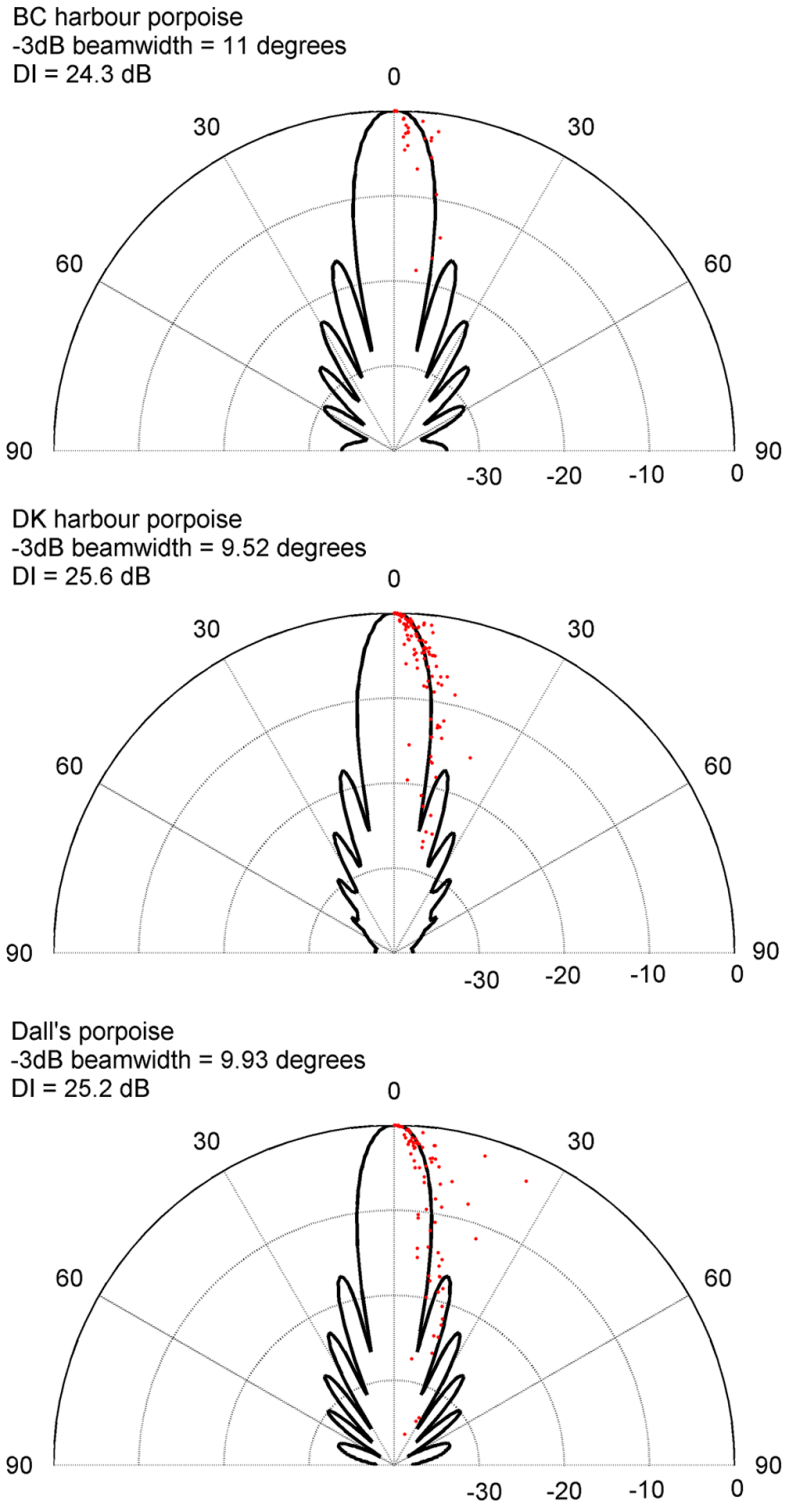
Vertical transmission beam patterns of Canadian harbour porpoise, Danish harbour porpoise and Dall’s porpoise. A) Canadian harbour porpoise, b) Danish harbour porpoise and c) Dall’s porpoise. The points are field data. On-axis clicks recorded within 20 m from the array (5 BC harbour porpoise, 19 Danish harbour porpoise, 15 Dall’s), each with the five off-axis versions recorded on the other hydrophones simultaneously. 0–90 are degrees off-axis re on-axis at 0°. 0 to −30 is dB re on-axis source level.

Composite, vertical beam patterns of the three porpoise species are shown in figure 4. The beam patterns were built only on clicks recorded within very short range (<20 m) of the vertical array and therefore only few clicks were available for each species: 5 BC harbour porpoise clicks, 19 Danish harbour porpoise clicks and 15 Dall’s porpoise clicks. Canadian harbour porpoises had equivalent transmission apertures [3] of 11.0 cm, Dall’s porpoise of 9.9 cm, while the Danish harbour porpoises had the smallest equivalent aperture of 9.5 cm (Figure 4). The directivity index (DI) was correspondingly highest for Danish harbour porpoises of 26 dB. Dall’s had a similar high DI of 25 dB, while Canadian harbour porpoises had the lowest of 24 dB (Table 1). The estimated DIs are based on the assumption of a rotationally symmetric biosonar beam with identical horizontal and vertical beamwidth. However, it has been found that the biosonar beam pattern of harbour porpoises is slightly dorsoventrally compressed, with the vertical beam slightly narrower (−3 dB beamwidth of 11°) compared to the horizontal beam (−3 dB beamwidth of 13°) [5].

### Source Levels, Range and Frequency

**Figure 5:**
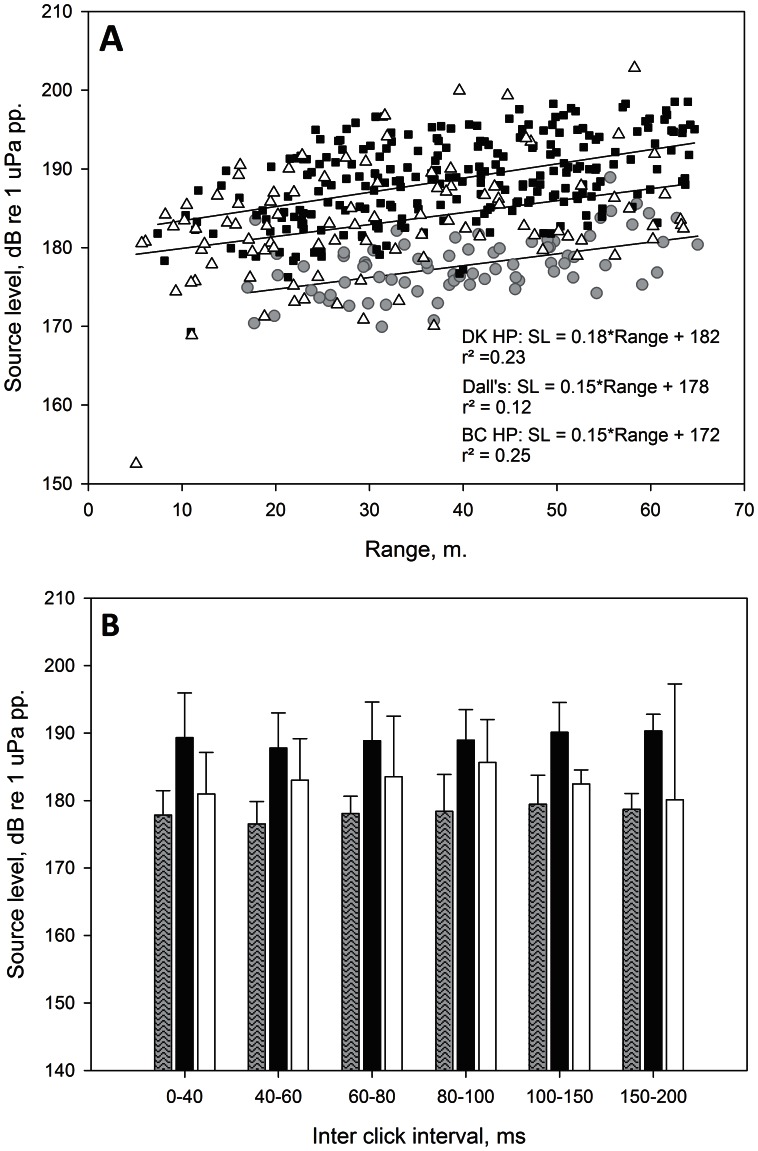
Source levels of Canadian harbour porpoises, Dall’s porpoise and Danish harbour porpoises. A) Source level plotted against range to array with linear regressions for Canadian harbour porpoise (BC-HP, grey circles), Danish harbour porpoise (DK-HP, black squares), Dall’s porpoise (Dall, white triangles). B) Mean source level and standard deviation per Inter-Click-Interval (ICI) band for Danish harbour porpoises (black), Canadian harbour porpoise (grey) and Dall’s porpoise (white). Danish harbour porpoises use clicks of significantly higher source level regardless of range or ICI band than the two other porpoise groups.

Source level varies as a function of click repetition rate/Inter-Click-interval (ICI) [32]. Source level may further vary with range, since range is used to back-calculate transmission loss and hereafter source level. Range may thus cause an artificial effect on source level. In order to test whether the source level differences observed among the three porpoise populations could derive from differences in inter-click-interval or recording range we made two tests. First we plotted source levels of the three porpoise types against recording range with regression lines and equation (Figure 5a). Secondly, we calculated mean source level as a function of inter-click-interval bands with standard deviation for each group (Figure 5b). The ICI bands were 0–40, 40–60, 60–80, 80–100, 100–150 & 150–200 ms, which were chosen to level sample size in each interval.

Figure 5a shows that the three groups were recorded at the same ranges from about 5 to 65 m. Source level did increase with range as expected, but with very low r^2^ values (Danish harbour porpoises r^2^ = 0.23, Canadian harbour porpoises r^2^ = 0.25 and Dall’s r^2^ = 0.12), and the differences in source level could therefore not be attributed different recording ranges. Figure 5b shows that within each porpoise group, the mean source level was not significantly different across the ICI bands (Kruskall Wallis, BC HP: *p = *0.512; DK HP: *p* = 0.439; Dall’s: *p* = 0.681). But when comparing the three porpoise groups, the source level was significantly different for each ICI band (Kruskall Wallis, *p*<0.001) except for 150–200 ms (Kruskall Wallis, *p* = 0.084). This means that the source level differences were independent of inter-click-interval in each species group and therefore that the source level differences reflect genuine population differences.

### Model of Target Detection Range for Signals of Different Centroid Frequency

**Figure 6:**
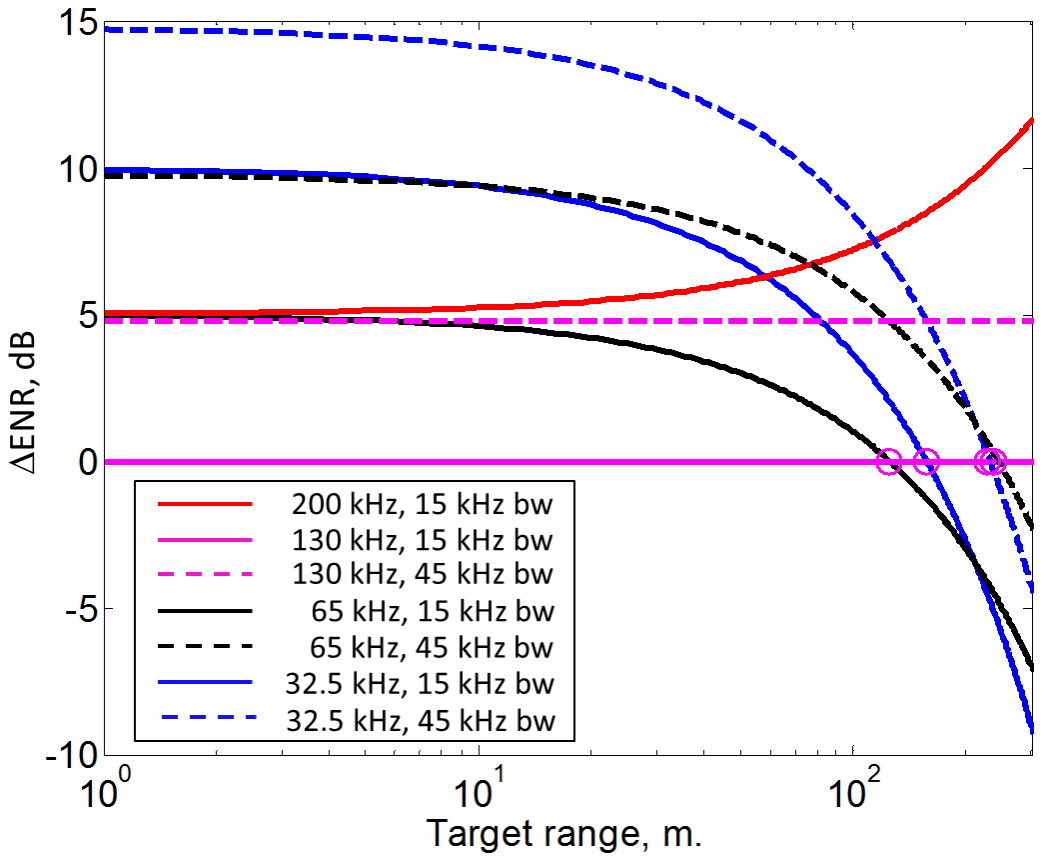
Effects of absorption and masking noise on detection range of NBHF and broadband dolphin style clicks. Two effects are modeled; 1) effects of varying centroid frequency (Solid lines) and 2) effects of increasing bandwidth three times (Broken lines). The effect is calculated as summed costs/benefit in dB in relation to the echo-detection- ratio (ENR) at detection threshold (DT) of a NBHF click (ΔENR) (y axis) with bandwidth of 15 kHz as a function of target range (x-axis). Thus at 0 dB the NBHF echo is just detectable for a given range, source level and target. A positive ΔENR value means that the animal would need to increase source level by that dB difference to detect that same target. If the ΔENR is below zero it means a better echo to noise ratio by that dB difference than for the NBHF signal. The figure assumes fixed source energy flux density level and target strength (TS). Masking noise levels (NL) are calculated from fig.7.5 in [2] for sea state 3 in deep water assuming a fixed bandwidth of 15 kHz. Absorption is calculated by equations given by [36] for relevant centroid frequencies (32.5, 65, 130 and 200 kHz) at 14°C and salinity of 33^o^/_oo_. Solid lines show detection ranges for a NBHF type click with varying centroid frequency. Broken lines of colour x mimics the effect of switching to a dolphin type click with a bandwidth of 45 kHz (3×NBHF bandwidth) with the same centroid frequency and source energy flux density as solid colour x. The figure shows that NBHF click yields longer detection ranges than dolphin type clicks out to ranges of about 200 m, assuming clicks of equal source energy flux levels. This is primarily caused by the lower detection threshold effects of a narrow bandwidth signal. (See full explanation and assumptions of the model in the methods section).

Results of the model are shown in Figure 6 as a function of target range (x-axis). Changes in echo-to-noise ratio (ΔENR) (y-axis) were normalized relative to the NBHF signal to aid interpretation. Thus at 0 dB in figure 6 the NBHF echo is just detectable for a given range, source level and target, so when a value ΔENR on the y-axis is greater than zero, it means that the animal would need to increase source level by that dB difference to detect that specific target. If the ΔENR is below zero it means a better echo to noise ratio by that dB difference than for the NBHF signal for the same source energy flux density, target and range.

The following was modeled (but see methods):

- *Absorption and spectral noise*. The effects of absorption on detection threshold was modeled for signals of different centroid frequencies (32.5, 65, 130 and 200 kHz) and equal bandwidths (15 kHz) and source energy flux densities. Effects of changes in centroid frequency on absorption and background noise as ΔENR (y-axis) and target range (x-axis) are shown as different colored solid lines, where y = 0 is the normalized NBHF signal.
- The model showed that for the same source energy flux density level and bandwidth, a click at 130 kHz would have the best echo to noise ratio for ranges shorter than some 100 meters compared to the other frequencies. Beyond target ranges of some hundred meters, for the same bandwidth, the absorption would render poorer echo to noise ratios compared to clicks at lower centroid frequencies, whereas a centroid frequency of 200 kHz would perform worse than NBHF clicks regardless of the range.
- *Bandwidth and masking noise*. Typical dolphin clicks have a much wider bandwidth than NBHF clicks do. Thus to test what effect a wider bandwidth has on detection range, bandwidth was assumed three times wider for the same four frequencies. Effects of increased bandwidth on ΔENR (y-axis) and target range (x-axis) are shown as different colored broken lines.
- The model shows that if the NBHF click is compared to a broadband dolphin click of the same centroid frequency and source energy flux density, but with a bandwidth three times wider, the echo to noise ratio for the same range is 5 dB worse for the broadband click. Broadband clicks of lower frequency (32.5 and 65 kHz), equal source energy flux density and three times wider bandwidth will first yield the same echo to noise ratio at target ranges of more than 200 meters. The NBHF click thus has an advantage compared to a broad band dolphin click in terms of detection range within some 200 m for the same source energy flux density.

## Discussion

The three porpoise populations recorded in this study made remarkably similar echolocation clicks across species and habitat (Figure 1, Table 1) corresponding closely to those previously recorded from these and other NBHF species [4], [13]–[15], [17]–[19]. However, for the first time, subtle differences were found between echolocation clicks of Dall’s and harbour porpoise occupying the same habitat, to the extent that Danish harbour porpoise clicks sound more like those of Dall’s porpoise than those of harbour porpoise in the Pacific.

Studies of harbour porpoises in captivity have shown that the spectral content of clicks changes slightly with click repetition rate and source level [32], and a first step in evaluating whether source parameters vary among different populations of porpoises should thus involve the level of variability of click source parameters within an individual animal. Within a click train, source levels decrease when repetition rates get high, likely because of restrictions in the pneumatic sound production apparatus [19], [32]. At the same time, a high source level is positively correlated with centroid frequency and negatively correlated with bandwidth. Spectral differences in content and source levels could thus result from comparing different modes of sonar outputs among the porpoise groups. This cannot be ruled out entirely here, but we strictly chose on-axis clicks for analysis according to five criteria, maximising chances that a click was recorded on-axis. This means that we purposefully only included the one click of maximum signal-to-noise ratio in a click train and omitted buzzes that are of a different mode and lower source levels. Since received signal-to-noise ratio depends on recording range, click trains of low source levels are typically not included unless recorded at very close range. This means that click trains of high source level likely are overrepresented in this dataset, because the porpoises did not come very close to the recording boat. Thus, in view of our conservative on-axis criteria it seems that at the same time we reduced the possible variation from intra-click train differences and we therefore find that the three datasets are collected under comparable conditions.

We hypothesized that there would be differences among the echolocation signals of sympatric porpoise species in British Columbia, and that there would be differences among populations of the same species in different habitats. However, when comparing the observed variation to the reported variability among non-NBHF dolphins [3], [40], [41] or among sympatric Microchiropteran bats [6]–[9] the recorded porpoise groups appear strikingly similar.

The most favoured explanation for the evolution of the NBHF clicks of porpoises, dolphins of the genus *Cephalorhynchus* and Kogiids (*Kogia* sp.) is that the click type evolved convergently as an acoustic crypsis against predation from killer whales [19], [23], [24] that cannot hear well above about 100 kHz [22]. Our results do not refute this theory, where the small body size of NBHF species have driven the peak frequency up, while the risk of predation have narrowed the bandwidth until the clicks were no longer audible to their predators [42]. The present study also supports the findings of [21] that porpoises have an extreme high pass filtering with no energy below 100 kHz, because none of the recorded porpoise clicks had energy below 100 kHz.

### Requirements of Operating an Effective Sonar System

A second striking result of this study is the consistent high directivity index (DI) of both porpoise species of about 25 dB (Figure 4). This DI is similar to some larger captive delphinids; 22.3–28.5 dB for false killer whale [43] and 25.4 dB for bottlenose dolphin [12] but smaller than for wild white beaked dolphin (*Lagenorhynchus albirostris*) with DI of 29 dB [44] and captive beluga whale (*Delphinapterus leucas*) with DI of 32.1 dB [3]. DI has been measured twice for captive harbour porpoises: first by Au and colleagues, who found a DI of 22 dB [39]; and later by Koblitz and colleagues, who found a DI of 24 dB [5]. The DI difference between the studies likely arose from methodological differences, since Au and colleagues [39] measured the vertical and horizontal beam pattern individually and averaged over many clicks from different angles, whereas Koblitz and colleagues [5] measured both the vertical and horizontal beam pattern simultaneously with an array of 16 hydrophones along four angles. This means that the original study [39] likely introduced some variation at each angle resulting in a lower DI. Here we calculated DI from individual clicks recorded simultaneously on seven different hydrophones. Because our results closely match results obtained with the 16 hydrophone array in captivity [5], we conclude that our methodology may likely accurately represent the DI of wild porpoises. We used the same methodology for similarly sized NBHF Commerson’s and Peale’s dolphins that also have DIs of 25 dB [4]. It therefore appears that a DI around 25 dB may be the minimum requirement for operating a functional biosonar in water, and that porpoises evolved their special echolocation click source properties to meet the dual requirements of operating an effective sonar system from a small head and at the same time to minimize the risk of killer whale predation from passive listening. The size of echolocating toothed whales thus ultimately defines frequency content through a minimum DI between 22 and 25 dB. However, since the signal will suffer from increasingly high absorption with increasing centroid frequency there is an upper limit on centroid frequency for efficient sonar, which may help explain that all recorded NBHF species have centroid frequency around 130 kHz [4], [14], [15], [17]–[19].

The opposing mechanisms of a need for high frequencies to make a directional sonar beam from a small head while facing high absorption at high frequencies, and risk of predation when phonating at lower frequency may thus have resulted in the narrow bandwidth of NBHF clicks around 130 kHz to which their hearing is matched. The mammalian auditory system is well modelled as a filter bank with overlapping frequency bands centred at different frequencies [4]. For mammals in general, the auditory filter bandwidth increases with centroid frequency to form a constant Q filter bank [3], [45]. However, the critical bands of finless and harbour porpoises only increase from 3 to 4 kHz when the centroid frequency increased from 32 kHz up to 140 kHz indicating that the porpoise auditory system is more accurately represented as a constant bandwidth filter bank [46]. With a constant Q-filter bank in white noise, masking noise increases with increasing centroid frequency, because the bandwidth increases. However, for a constant bandwidth filter bank, masking will decrease with increasing frequency in white noise [46] or normal ocean noise conditions thus providing an advantage.

### Auditory Advantages of the NBHF Click

We have illustrated the possible auditory advantages of the NBHF click in the model of Figure 6. First, the model shows that for the same source energy flux density level and bandwidth, a click at 130 kHz will have the best echo-to-noise ratio for ranges shorter than some hundred meters compared to the other frequencies due to the low noise window at 130 kHz [2]. The low noise window is found in deep water and low sea state and only assumed here for coastal waters as well, and this may well be too simplistic an assumption, but we lack the data to test it. Beyond target ranges of some hundred meters, for the same bandwidth, the absorption will render poorer echo-to-noise ratios for the NBHF click compared to clicks at lower centroid frequencies, whereas a centroid frequency of 200 kHz will result in poorer performance than a NBHF click regardless of the range.

Secondly the model shows that if the NBHF signal is compared to a broadband dolphin style signal of the same centroid frequency and source energy flux density, but with a bandwidth three times wider, it is seen that the echo-to-noise ratio for the same range is 5 dB worse at shorter ranges for the dolphin style click. It will only yield the same echo-to-noise ratio at target ranges of more than 200 meters. Yet, if the bandwidth of a 130 kHz signal is three times wider the low-noise window only offers a small advantage in detection range in relation to the lower centroid frequency signals at 15 kHz bandwidth, and only for very short ranges. Thus, for a fixed source energy flux density level, the low noise window at 130 kHz offers an advantage of the NBHF signal out to about 100 m, while the narrow band properties offers an advantage out to about 200 m at 130 kHz. The narrow band signal is as such very well suited for short-range sonar. This then raises the question of why all delphinids do not use very narrow band signals at different centroid frequencies depending on their size and needs of sonar ranges. Due to a shorter click duration, a dolphin species will require 3–6 dB more pressure to generate the same energy flux density as NBHF species, so if all toothed whales were peak pressure limited, NBHF species would have an overall advantage due to their narrow bandwidth, long duration and the low-noise window around 100–150 kHz. However, if production of NBHF clicks somehow is peak pressure limited (some 200 dB re 1 uPa (p-p) according to all available data [4], [15], [18], [19], [47] compared to similar sized dolphin species (SL up to 225 dB re 1 uPa (p-p), [3], [48], it seems that dolphin species can overcome the 3–6 dB difference *and* the 5–15 dB poorer echo-to-noise ratio from a larger masking bandwidth simply by creating a higher peak pressure. So if the long duration of NBHF clicks should serve to increase energy flux density [3], why not just make normal broadband dolphin clicks, where the energy goes up with the square of the pressure (i.e. 6 dB more source energy would either require four times longer duration or twice the pressure)? This may be answered by the anti-predation theory that requires clicks produced without energy at frequencies below 100 kHz. Since the model, with its inherent limitations due to the many assumptions, shows that there are no overall advantages of the NBHF click compared to dolphin clicks produced with a higher source level, it is implied that the long duration of NBHF clicks has evolved to generate a narrow bandwidth as opposed to increasing energy flux density as proposed by Au [3]. The sonar requirements may thus have driven the selection for the high centroid frequency required for a high DI, whereas the bandwidth has been narrowed subsequently to reduce risk of predation from killer whales.

### Character Displacement

Given the above selection pressures for obtaining a functional biosonar and remaining inaudible to killer whales, the similarity among the recorded porpoise groups is less surprising and species and habitat differences must inherently be small. We hypothesized that the two sympatric porpoise species should have different click source properties to reinforce speciation rather than hybridization. Such acoustic differences are potentially important cues for finding the right species in dark waters beyond the short distances where vision may be used. We do in fact observe subtle, but significant differences in centroid frequency between sympatric Dall’s and harbour porpoises in British Columbia (BC) (Table 1). The mean centroid frequency of harbour porpoises was about 4 kHz higher than Dall’s porpoises in BC, while the Danish harbour porpoises were more similar in centroid frequency to Dall’s porpoise than to their BC conspecifics (Figure 2). Since the difference in centroid frequency seemingly is area-dependent rather than species-dependent it may be an example of character displacement [49] to enable acoustic species recognition in two closely related sympatric species producing otherwise very similar clicks. We found a similar 4 kHz difference in centroid frequency between two closely related sympatric NBHF dolphins at the Falkland Islands [4]. The question then is whether porpoises are in fact able to hear such a 4 kHz difference and whether hybridization is or has been a problem in terms of wasted reproductive effort [50]?

One line of evidence supporting the notion that the 4 kHz click frequency difference is large enough to be exploited for species differentiation is that this difference matches the auditory filter size of 4 kHz [46], [51]. However, there appears to be a mismatch between click centroid frequency and frequency of best hearing for harbour porpoises based on the available audiograms [37], [38]: One would expect frequency of best sensitivity to be equal to the centroid frequency of the emitted clicks, however the frequency of best hearing is around 100 kHz and not 130 kHz [37], [38]. To mimic the situation of a poorer sensitivity at the centroid frequency, we filtered clicks from both Dall’s and harbour porpoises with the harbour porpoise audiogram [37], [38] and calculated new source parameters for the filtered clicks. The filtered clicks were then submitted to a Monte Carlo simulation to see if the differences in centroid frequency were persistent and still large enough for species separation. The results in fact yielded a better discrimination potential than before the filtering (Figure 3) and the BC species could easily be separated. This points to the differences in the frequency centroid between species to be based in differences in the lower cut-off frequency of the clicks, and this strengthens the idea that the animals themselves can perform species differentiation based on the 4–5 kHz centroid frequency differences.

Frequency difference limens has not been assessed for porpoises, however bottlenose dolphins have a very acute frequency discrimination and can separate tones of only 1 kHz difference around 130 kHz [52]. A porpoise, with narrower auditory filters around this frequency, would be expected to at least equal, if not exceed, the bottlenose dolphin in differentiating between frequencies. That porpoises may have a similar fine frequency resolution is also suggested by the observation that a captive harbour porpoise accurately (>90% correct) could distinguish between balls of equal size but of different material (brass, steel, pvc, plexiglass) when blindfolded [53]. This capability would hence suggest that they can also separate between clicks from different species with mean differences of 4 kHz.

That hybridization actually occurs between porpoise species is evident in British Columbia from numerous strandings and observations of animals intermediate in shape and coloration [54], [55]. It is consistently harbour porpoises that seem to father the hybrids and it has been speculated that since the harbour porpoise population is declining in the area, male harbour porpoises, with their extremely large testes [56] and promiscuous behaviour, may be driving the hybridization through indiscriminate pursuit of females of either species [55]. Such behaviour cannot be prevented by accurate species discrimination.

Differences in source levels on other hand may be caused by environmental factors rather than species differences. In the present study, Danish harbour porpoises used clicks of significantly higher source level than both BC harbour and Dall’s porpoises (Figure 5), despite that Dall’s porpoise also produced few clicks of equally high source level showing they are capable of producing the same output as the smaller harbour porpoise. The source levels could be caused by differences in prey type, i.e. target strength, clutter levels or ambient noise levels. Unfortunately, we did not measure either during the field efforts and we cannot qualify the previous notions.

### Passive Acoustic Monitoring

The documented spectral species differences also have important implications for passive acoustic monitoring. If dataloggers with sufficient frequency resolution are used, there is an acoustic basis for differentiating between several sympatric NBHF species, provided that similar species differences exist. Since acoustic monitoring is a cheap alternative to visual monitoring, and since many NBHF species are subject to high bycatch rates (e.g. [57]) it seems relevant to pursue this possibility in the near future.

### Conclusion

We conclude that porpoises produce clicks of high directionality that is comparable to that of larger dolphins, and we argue that the click source parameters of porpoises likely evolved to meet the dual requirements of operating an effective sonar system from a small head and at the same time to minimize the risk of killer whale predation from passive listening. Within these constraints there appear to be little more than a few kilohertz at play for species differences and habitat specializations, and only at the low-frequency cut-off. The observed spectral species differences likely evolved as a character displacement, a prezygotic barrier to obtain reproductive isolation of two sympatric species and thereby reduce the risk of hybridization, but may as well be utilised for species separation in passive acoustic monitoring.
